# Hemodynamic deterioration precedes onset of ventricular tachyarrhythmia after Heartmate II implantation

**DOI:** 10.1186/s13019-016-0493-0

**Published:** 2016-07-08

**Authors:** Ameeta Yaksh, Charles Kik, Paul Knops, Korinne Zwiers, Maarten J. B. van Ettinger, Olivier C. Manintveld, Marcel C. J. de Wijs, Peter van der Kemp, Ad J. J. C. Bogers, Natasja M. S. de Groot

**Affiliations:** Unit Translational Electrophysiology, Department of Cardiology, Erasmus Medical Center, PO Box 616, ‘s Gravendijkwal 230, 3015CE Rotterdam, The Netherlands; Department of Cardiothoracic Surgery, Erasmus Medical Center, Rotterdam, The Netherlands; Sorin, Rotterdam, The Netherlands

**Keywords:** Heartmate II, Continuous rhythm monitoring, Ventricular tachycardia, Hemodynamics

## Abstract

**Background:**

Early postoperative ventricular tachyarrhythmia (PoVT) after left ventricular assist device (LVAD) implantation are common and associated with higher mortality-rates. At present, there is no data on initiation of these PoVT and the role of alterations in cardiac hemodynamics.

**Case Presentation:**

A LVAD was implanted in a patient with end-stage heart failure due to a ischemic cardiomyopathy. Alterations in cardiac rhythm and hemodynamics preceding PoVT-episodes during the first five postoperative days were examined by using continuous recordings of cardiac rhythm and various hemodynamic parameters. All PoVT (N=120) were monomorphic, most often preceded by short-long-short-sequences or regular SR and initiated by ventricular runs. Prior to PoVT, mean arterial pressure decreased; heart rate and ST-segments deviations increased.

**Conclusions:**

PoVT are caused by different underlying electrophysiological mechanisms. Yet, they are all monomorphic and preceded by hemodynamic deterioration due to myocardial ischemia.

## Background

Early postoperative ventricular tachyarrhythmia (PoVT) after implantation of left ventricular assist devices (LVAD) are common, despite the unloading of the left ventricle, and are associated with higher mortality-rates due to eventually failure of the right ventricle [1–4]. The aetiologies of VT’s after LVAD implantation are multifactorial, e.g. hypovolemia [3] and electrolyte imbalance [5]. Knowledge of PoVT-onset, might provide insight into the mechanism underlying PoVT and thereby provide development of preventive measurements. Previous studies suggested that variations of rhythms preceding VT (e.g. short-long-short sequences (SLS-sequences)) and modes of initiation (e.g. ventricular premature beats (VPB) or sudden onset (SO)) imply different mechanisms underlying VT, including re-entry, triggered activity or abnormal automaticity [6, 7]. In patients with various types of cardiomyopathies and implantable cardioverter-defibrillators (ICD) rhythm preceding VT were studied by using intracardiac electrograms. In some patients, VT were initiated by VPBs whereas in other patients VT started suddenly [8–13]. Differences in VT onset were not associated with the underlying cardiac disease. VT’s initiated by VPBs had shorter cycle lengths (CL) compared to VT’s starting suddenly [8–13]. VPB induced VT were more often preceded by SLS-sequences than SO VT [8–10, 12]. In patients with preserved systolic function, the majority of the VT’s had a SO [8–10]. Analysis with 24-h Holter in patients silent ischemia demonstrated that recurrent monomorphic VT’s were often preceded by SLS-sequences and subsequently induced by VPBs [14]. Several studies reported on the occurrence of VT early after Heartmate II implantation but the onset of these PoVT has not yet been examined [15–19]. In addition, the role of alterations in cardiac hemodynamics in initiation of PoVT has also not been investigated. In this case report, we describe rhythm characteristics and hemodynamic alterations preceding PoVT-episodes during the first five days after implantation of the Heartmate II by using continuous recordings of cardiac rhythm and hemodynamic parameters.

## Methods

This case report is part of the Rotterdam Rhythm Monitoring Project (AMOR) (approved by the institutional medical ethical committee (MEC 2012-481)). Written informed consent was not applicable. Continuous postoperative rhythm recordings were semi-automatically analysed for the first five days after Heartmate II implantation. PoVT-episodes (series of ventricular beats lasting ≥30 s) were classified as either monomorphic or polymorphic; CL of every PoVT-episode was assessed separately. Using 5 beats preceding a PoVT-episode, rhythms were subdivided into 1)sinus rhythm (SR), 2)SR with ventricular-run (V-run) or 3)SLS-sequences. PoVT-episodes were initiated by either a VPB, ventricular-couplet (V-couplet) or -run, or it started with a SO. In the latter case, the morphology of the first PoVT beat was similar to the remainder of the PoVT beats and the prematurity index (PI) of the first beat had to be <110 % [9]. The PI of the first beat was calculated as the ratio between the CL of the first and second beat of the PoVT. PoVT with a PI <110 % was classified as SO and the PI was >110 % it was a VPB-initiated PoVT [8, 9, 11, 13]. In order to examine alterations in cardiac hemodynamic, right intra-atrial pressure (RAP), mean arterial pressure (MAP), heart rate (HR) and ST-segment deviation (d-ST) prior to PoVT-episodes were obtained 30 s and 1 min preceding every PoVT-episode and compared with data obtained from similar time intervals at the start of the rhythm registrations, the so-called reference-data.

### Statistical analysis

Continuous normally distributed variables were expressed as mean ± SD. The unpaired, two tailed Student’s *T*-test was used to demonstrate differences between continuous variables. Mann-Whitney *U*-test was applied for continuous skewed data. A *P*-value of <0.05 was considered statistically significant.

## Case presentation

**Fig. 1:**
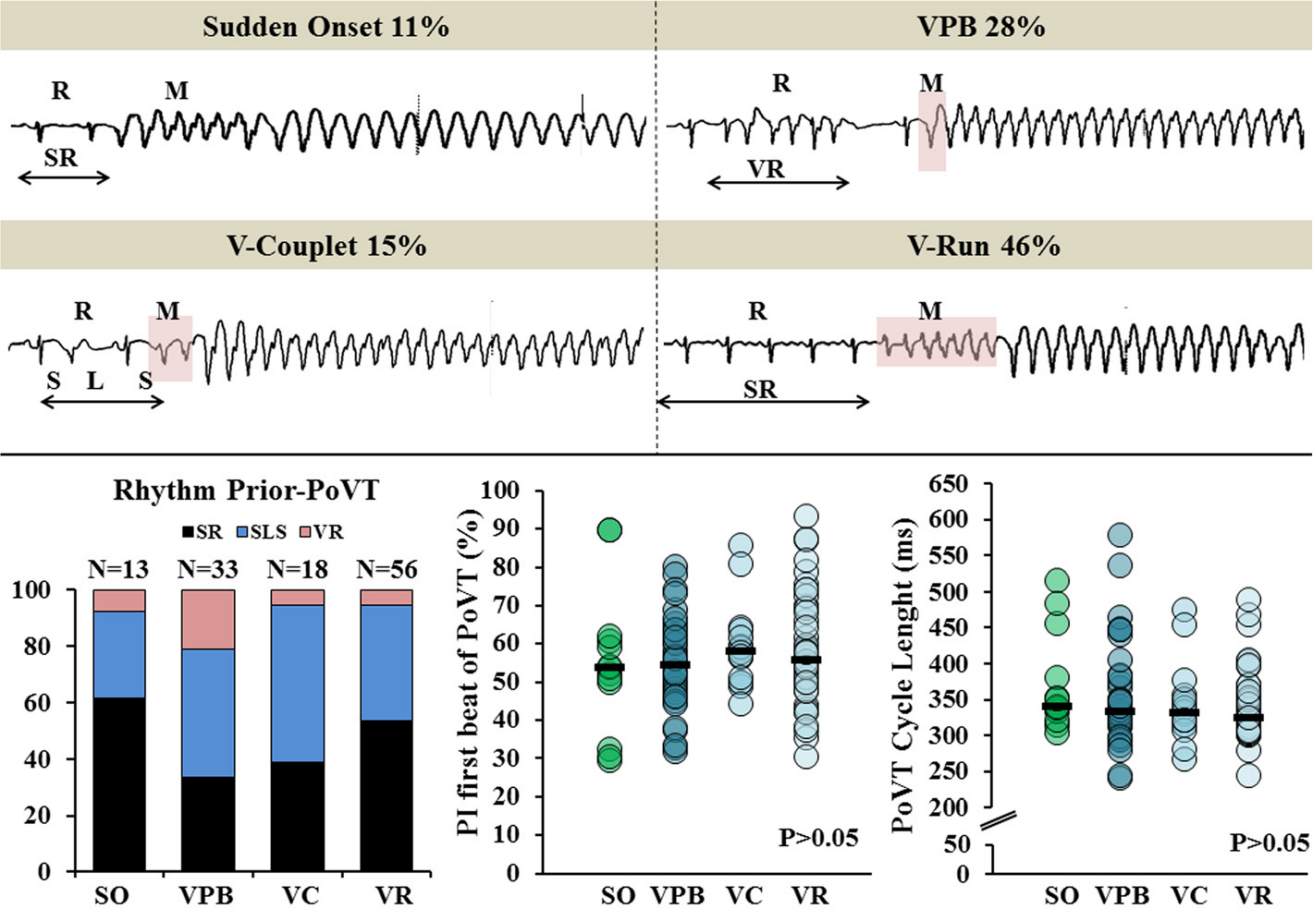
Initiation characteristics of postoperative VT. Upper panel: examples of the four different VT-onset modes with different preceding rhythms. Lower panel: characteristics of rhythm preceding postoperative VT. See text for detailed explanation. SO = sudden onset, VPB = ventricular premature beat, VC/R = ventricular couplet/run, SR = sinus rhythm, SLS = short-long-short sequence, PI = prematurity index

A 47-year-old male with end-stage heart failure due to ischemic heart disease received a Heartmate II (a rotary continuous flow device) which served as a bridge-to-heart transplantation [20]. Prior to surgery, no conduction disorders were present, therapy consisted of anti-arrhythmic agents class II and III and an ICD was not implanted. An circular incision in the apex of the left ventricle, to implant the outflow cannula, was made by a circular sharp object (provided by the LVAD company). This circular object was positioned parallel to the ventricular septal wall and opposite to the mitral valve in order to achieve optimal blood flow into the LVAD. After completion of the incision a ring sutured inside (airing) and the outflow cannula was positioned and the LVAD was filled. Finally, the inflow cannula was connected to the aorta ascendens. The procedure was performed on beating hart with temporary support of the cardiopulmonary bypass (lasting one hour and 27 min). During the first five days after Heartmate II implantation 120 monomorphic PoVT-episodes occurred with an average duration of 14 ± 77(0.5–842)min (total burden: 32.6 %). PoVT-episodes were preceded by either regular SR (*N* = 55, 46 %), SLS-sequences (*N* = 52, 43 %) or V-run (*N* = 13, 11 %) and started either suddenly (*N* = 13, 11 %), with a VPB (*N* = 33, 28 %), V-couplet (*N* = 18, 15 %) or V-run (*N* = 56, 46 %) as is depicted in Table 1; examples are illustrated in the upper panel of Fig. 1. The averaged median CL of the 5 beats preceding PoVT was 595 ± 87 ms and did not differ between PoVT-episodes with different modes of onset (median CL SO:639 ms, VPB:571 ms, V-couplet:587 ms, V-run:601 ms (*P* > 0.05)). In case of regular SR preceding PoVT, the averaged CL of the beats preceding PoVT were significantly shorter compared to the averaged CL of the beats in the reference time periods (590 ms versus 732 ms; *P* < 0.001). The left lower panel of Fig. 1 illustrates that PoVT-episodes starting suddenly or with a V-run were mainly preceded by SR (respectively 62 % (*N* = 8) and 52 % (*N* = 29)), whereas PoVT with VPBs or V-couplets at the onset are mostly preceded by SLS-sequences (respectively 44 % (*N* = 15) and 56 % (*N* = 10)). For every type of PoVT-onset, there was a large variation in the PI, but there were no significant differences in median PI between the various onset categories (SO:55 %, VPB:55 %, V-couplet:59 % and V-run:58 % (*P* > 0.05), as demonstrated in the middle lower panel of Fig. 1. The lower right panel of Fig. 1 demonstrates no differences in the median PoVT CLs of the different PoVT-onsets (SO:372 ms, VPB:356 ms, V-couplet:343 ms and V-run:338 ms (*P* > 0.05)).

**Table 1:** Various rhythm preceding PoVT and several modes of onset

		Rhythm prior to PoVT		
		Sinus Rhythm	Short-long-short-sequence	Ventricular run with sinus rhythm beats
Mode of PoVT onset	Sudden onset; *N* = 13; 11 %	*N* = 8; 61 %	*N* = 4; 31 %	*N* = 1; 8 %
	Ventricular premature beat; *N* = 33; 27 %	*N* = 11; 33 %	*N* = 15; 46 %	*N* = 7; 21 %
	Ventricular Couplet; *N* = 18; 15 %	*N* = 7; 39 %	*N* = 10; 55 %	*N* = 1; 6 %
	Ventricular run; *N* = 56; 47 %	*N* = 29; 52 %	*N* = 23; 41 %	*N* = 4; 7 %

**Fig. 2:**
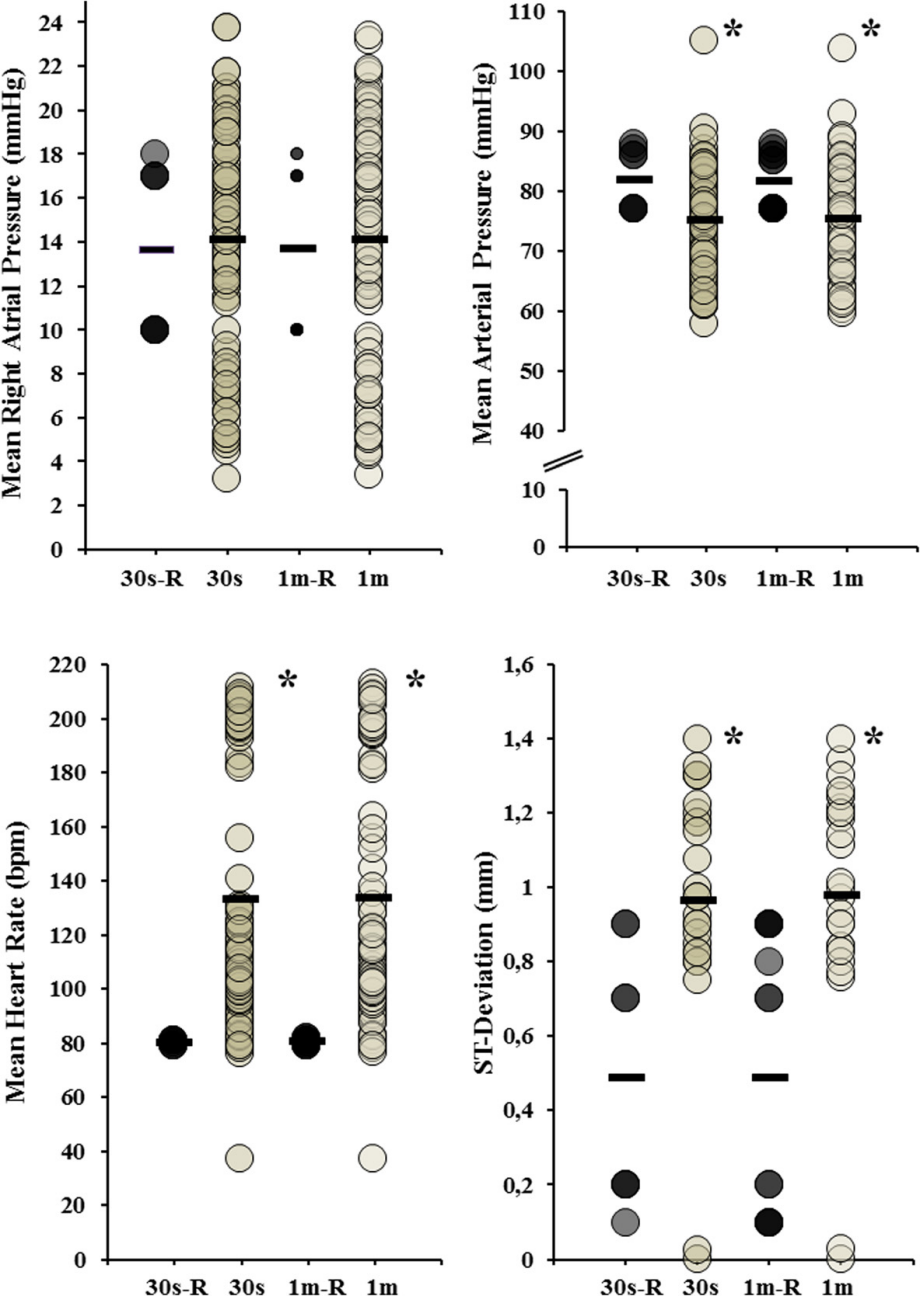
Postoperative VT: hemodynamic characteristics. Hemodynamic parameters, including right atrial pressure (RAP), mean intra-arterial pressure (MAP), heart rate (HR) and absolute ST-segment deviation of lead II (d-ST), in 30 seconds (30s) and 1 minute (1 m) before PoVT-episodes are compared to the similar time intervals at the reference time period (respectively 30s R and 1 min R). *P < 0.05. R = reference time interval

Differences between hemodynamic parameters (RAP, MAP, d-ST and HR) 30s and 1 min. prior to every PoVT-episode in comparison with similar reference-data are illustrated in Fig. 2. Averaged MAP 1 min and 30 s. preceding PoVT-episodes decreased; both 72 mmHg versus 82 mmHg (*P* = 0.008 and *P* < 0.001, and d-ST and HR were higher; both time intervals 1.0 mm versus 0.5 mm (*P* < 0.001); 133/bpm and 134/bpm both versus 80/bpm (*P* < 0.001). RAP did not alter prior to development of PoVT-episodes. The patient finally died 13 days after Heartmate II implantation due to incessant VTs.

The LVAD rotational speed during the postoperative period was around 9200 rpm with an mean flow rate of 5.2 L/min, which indicates no major suction events. Prior to death, both rotational speed and flow decreased.

## Discussion

In our patient, all PoVT-episodes were monomorphic. The phenomenon of similar monomorphic episodes in one patient has previously also been defined as intra-individual reproducibility (80–88 %) [8, 9]. This observation suggests that either the re-entrant circuit or site of ectopic activity is the same for every PoVT-episode in case of respectively re-entry or focal activity as the underlying mechanism. Rhythms prior to VT’s were most often SLS-sequences or SR comparable to observations made by Gomes et al in patients with silent ischemia [14]. However, the multiple types of VT-initiation in this study reflect involvement of different underlying mechanisms in PoVT-onset [14].

Of interest is that all PoVT-episodes in our patient are preceded by a significant decrease in MAP, an increase in d-ST and HR suggesting that decreased myocardial perfusion plays an important role in the pathophysiology of PoVT. The resulting myocardial ischemia in turn may induce heterogeneity in conduction, dispersion in refractoriness or enhance ventricular ectopy. In addition, ventricular ectopy is caused by e.g. triggered activity or enhanced automaticity due to on-going increased ventricular stretch in the postoperative period. In the presence of an extensive substrate due to myocardial scarring (rarely caused by the site of the inflow cannulation), VT may be initiated by a trigger [3]. As described previously [3, 4], changes in hemodynamics may also be the result of adaptation to LVAD function due to changes in pre- and afterload [3, 4]. It is very difficult to distinguish whether these hemodynamic changes in our patient were due to changes caused by the LVAD or consequences of the VTA. However, there was were no obvious changes in the LVAD rotational speed and flow in our patient. Neither did we observe major changes in electrolytes during the postoperative period. This in contrast with Monreal et al. [5] who reported acute alterations in myocardial electrolytes in sheeps supported with LVAD. These results might only partially apply in humans.

As VTs are the most common tachyarrhythmia occurring after implantation of LVADs (early- and late-postoperative VTs ranged from respectively 13–28 % and 11–66 %) [3, 4, 15, 18, 21] and are associated with higher mortality-rates (early-postoperative 54 % versus late-postoperative 10 %, *P* < 0.01) [1] preventive measures are essential. The observations in our patient indicate that early detection of hemodynamic alterations followed by corrective measures might prevent development of PoVT and in turn decrease mortality in patients undergoing implantation of a LVAD.

## Conclusion

PoVT-episodes in a patient with ischemic cardiomyopathy after Heartmate II implantation are induced by different electrophysiological mechanisms though the resulting PoVT are monomorphic. All PoVT-episodes were preceded by hemodynamic deterioration indicating that myocardial ischemia is the major contributor to development of PoVT. Hence, optimization of cardiac hemodynamics is essential for prevention of PoVT-episodes.

## Abbreviations

CL: Cycle length
d-ST: ST-segment deviation
HR: Heart rate
ICD: Implantable cardioverter-defibrillator
LVAD: Left ventricular assist device
MAP: Mean arterial pressure
PI: Prematurity index
PoVT: Postoperative ventricular tachyarrhythmia
RAP: Right intra-artrial pressure pressure
SLS-sequences: Short-long-short sequences
SO: Sudden onset
SR: Sinus rhythm
V-couplet: Ventricular-couplet
VPB: Ventricular premature beat
V-run: Ventricular-run
